# Bioactivity of Encapsulated Lemon Peel Phenolics as Affected by Maltodextrin and Foam Mat Drying

**DOI:** 10.1002/fsn3.70595

**Published:** 2025-07-14

**Authors:** Nihal Durmus, Meral Kilic‐Akyilmaz

**Affiliations:** ^1^ Department of Food Engineering Istanbul Technical University Istanbul Türkiye; ^2^ Department of Food Processing Akcakoca Vocational School, Duzce University Duzce Türkiye

**Keywords:** angiotensin‐I‐converting enzyme, drying, lemon peel extract, stability, α‐amylase

## Abstract

Lemon peel constitutes a cheap potential resource for bioactive phenolics with antioxidant, antihypertensive, and antidiabetic activities. However, phenolic compounds are sensitive to environmental conditions and lose their activity during processing and storage without protection. In this study, a phenolic extract obtained from lemon peel was encapsulated to protect its bioactivity. For this purpose, two maltodextrins with 6‐ and 19‐dextrose equivalent (6DE, 19DE) and whey protein concentrate were used along with foam mat drying (FMD) and foam mat freeze‐drying (FMFD). The effects of maltodextrins and drying method were evaluated by determination of encapsulation efficiency, physicochemical properties, in vitro antihypertensive, and antidiabetic activities of the encapsulated extracts. In addition, encapsulated extracts were stored under adverse conditions to determine storage stability. All the encapsulated extracts were found to be in a glassy state. Maltodextrin with 6DE was the best wall material protecting the phenolic content. FMD with maltodextrin 6DE provided the highest encapsulation efficiency (97.6%) and hesperidin content (45 mg/100 g dm). On the other hand, FMFD with maltodextrin 6DE resulted in the highest glass transition temperature (97.1°C), total phenolic content (6.63 mg GAE/g), and antioxidant activity (17.6 mg TE/g). ACE inhibitory activities of the encapsulated extracts were above 75% but reduced to less than 50% after storage regardless of maltodextrin DE and the drying method. FMFD with maltodextrin 19DE yielded 71% α‐amylase inhibitory activity, which was higher than those of the other samples. α‐amylase inhibition increased up to 83% after storage with no significant effect of maltodextrin DE and drying method. Results showed that the wall materials used in encapsulation also have an influence on the bioactivities besides the lemon peel extract. Both FMD and FMFD along with maltodextrin 6DE can be used for encapsulation. However, FMD can be preferred as a practical method with low equipment and energy costs and short processing time.

## Graphical abstract

Lemon peel phenolic extract with antihypertensive and antidiabetic bioactivity was encapsulated with WPC and maltodextrin by foam mat drying. Foam mat drying can be preferred as a practical method with low equipment and energy cost and short processing time. The wall materials also contributed to the measured bioactivities besides the lemon peel extract.

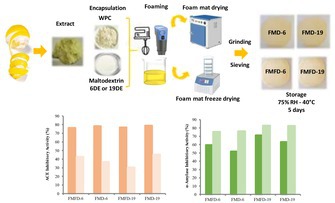

## 1 Introduction

Lemon ( *C. Limon* L.) is one of the most popular fruits among *Citrus* species belonging to the *Rutaceae* family. Processing of lemon creates a large quantity of waste including peels, seeds, and pulps weighing around 50% of the whole fruit. Peel constitutes the major portion of lemon waste, about 30%–65% of the fruit, which is a potential resource for valuable food components such as pectin and phenolic compounds (Magalhães et al. 2023). Peels consist of two layers; an exterior layer called the epicarp or flavedo and an interior layer called the mesocarp or albedo. The flavedo is rich in phenolic compounds, mainly flavonoids such as hesperidin, diosmin, eriocitrin, and narirutin, whereas the albedo is rich in dietary fibers such as pectin (Magalhães et al. 2023). Phenolics in lemon peel waste can be recovered and reutilized as an upcycling strategy. However, phenolic compounds are susceptible to oxidation, which can occur as a result of exposure to oxygen, heat, and light (Papoutsis et al. 2018). Therefore, they need to be protected during processing and storage until consumption. Encapsulation is a technique in which selected active compounds can be enclosed in a matrix to preserve them from degradation and oxidation during storage and passage through the gastrointestinal system. There are several techniques for encapsulating bioactive compounds. Process techniques used for encapsulation in the food industry include coacervation, spray drying, extrusion, spray chilling, liposome entrapment, fluidized bed coating, inclusion complexation, and freeze‐drying (Jia et al. 2016; Joye and McClements 2014).

Freeze‐drying is widely employed in the encapsulation of bioactive extracts due to equipment availability, simplicity, and desired end product qualities (Kandasamy and Naveen 2022). Freeze‐drying does not employ flowing hot air like convective drying; therefore, the risks of thermal and oxidative damage are negligible. The resulting powder is porous and has high bioactive retention, improved rehydration ability, and minimal nutritional loss (Farahmandfar et al. 2020). Although freeze‐drying has been acclaimed for producing high‐quality food powders, it has been considered expensive due to significant equipment investment, running costs, and longer dehydration times.

Drying can also be facilitated through a foam matrix for food materials as a cost‐effective alternative. When a foam of liquid or semiliquid material spread as a thin layer on a mat or tray is dried by conventional hot air drying, the drying method is called foam mat drying (FMD). Foaming agents and foam stabilizers such as whey protein isolate (WPI), sodium caseinate, egg white, and methylcellulose are added to the material before whipping, bubbling, or shaking the mixture. The greater surface area available in FMD is a significant advantage which enhances the drying rate, resulting in a decrease in drying time and processing cost (Hardy and Jideani 2017; Kanha et al. 2022). To avoid the drawbacks of conventional hot air drying, foam mat freeze‐drying (FMFD), which combines foaming and freeze‐drying, is being investigated as a cost‐effective alternative (Hardy and Jideani 2017). FMFD has been shown to increase the drying rate, glass transition temperature (Tg) of the matrix, and storage stability compared to freeze‐drying (Rin‐ut and Rattanapitigorn 2020). FMFD has been successfully used for the drying of blueberry juice (Darniadi et al. 2019).

Maltodextrin and whey proteins have been commonly used for encapsulation of various bioactive materials to improve drying efficiency and stability. They function by increasing solid content, viscosity, and chemical and physical interactions, entrapping the target compounds. Maltodextrins derived from starch are preferred due to their low cost, availability, water solubility, and film‐forming capability. Functional and physicochemical properties of a maltodextrin are dependent on the dextrose equivalent (DE) value that indicates the level of starch degradation. High DE maltodextrins have higher hygroscopicity, permeability, solubility, sweetness, and reactivity in the browning reaction, while they result in lower Tg, more freezing point drop, and lower stability, viscosity, and resistance to crystallization (Xiao et al. 2022). Although maltodextrin is a good wall material, it may rapidly release the inner bioactive core during digestion, leaving bioactives exposed to severe gastrointestinal conditions (Rashid et al. 2022). Hence, whey proteins have been frequently used in combination due to the additional protection provided through their interactions with the bioactive compounds, emulsifying, foaming, and gelling abilities. The mixture of wall materials provides protection mostly through hydrogen bonds that are formed with polysaccharides and proteins when water is removed during the drying process.

Handling and drying of fruit extracts are difficult as they are sticky due to their high sugar and acid content. In addition, encapsulation is required for protection of bioactive components during processing, storage, and digestion. FMD with wall materials can be a low‐cost technique that allows efficient encapsulation and drying of such food materials. Although process optimization studies have been carried out for FMD (Gaikwad et al. 2024), studies on its effect on bioactive properties of the food materials are scarce. The objective of this study was to investigate the use of maltodextrins with 6‐ and 19‐dextrose equivalent (6DE, 19DE) and whey protein concentrate (WPC) as wall materials along with FMD and FMFD for encapsulation of lemon peel phenolic extract.

## 2 Materials and Methods

### 2.1 Materials

Lemons of Lamas cultivar were supplied from a local producer (Antalya, Türkiye). Lemon peels were cut into rectangular pieces and freeze dried (Labconco Freezone 2.5 Plus, Kansas City, MO, USA) at 0.065 mBar and −48°C for 48 h. A powder was obtained after grinding dried peels with a stainless‐steel grinder (Bosch MKM6000, Slovenia) and screening through a metal sieve with a pore diameter of 450 μm. A plant tissue‐degrading enzyme, lysing enzyme from *Aspergillus* sp., containing cellulase and pectinase (L3768, Sigma‐Aldrich, St. Louis, MO, USA) was used for extraction. Two maltodextrins with 6DE and 19DE (Glucidex, Roquette Frères, Lestrem, France) and WPC (MilkPro, Slava Süt, Konya, Türkiye) were used as wall materials for encapsulation. WPC contained 70% protein in dry matter.

### 2.2 Extraction of Phenolic Compounds

Phenolic compounds were extracted from 1 g of lemon peel powder by enzyme‐assisted extraction using the enzyme (5 U) in 20‐mL acetate buffer (20 mM, pH 5.0) at 40°C for 60 min (Ruviaro et al. 2019). The mixture was cooled in an ice‐water bath after incubation. The hydrolysate was mixed with 20 mL of 50% aqueous ethanol on a magnetic stirrer at a rate of 150 rpm at 23°C for 40 min. Supernatant containing the phenolic compounds was separated by centrifuging at 2700 × g for 20 min at 4°C. The supernatant was freeze dried as explained above. The dried extract was ground and sieved through a screen with a pore diameter of 200 μm.

### 2.3 Encapsulation

Lemon peel phenolic extract powder was encapsulated by using two maltodextrins with 6DE or 19DE together with WPC. Maltodextrin and WPC at a mass ratio of 1:1 were prepared at a total concentration of 15% (w/v) in water and mixed with 1 g of extract (Papoutsis et al. 2018). The mixture was whipped with a hand mixer at a speed of 1000 rpm for 3 min and then homogenized using a probe homogenizer (Ultra‐Turrax T18 Basic, IKA‐Werke GmbH & Co. KG, Staufen, Germany) at 10000 rpm for 1.5 min. In the foam mat drying process (FMD), foamed samples were placed in a dish and dried in an oven at 50°C until obtaining a constant mass. In the foam mat freeze‐drying process (FMFD), foamed samples were initially frozen overnight at −20°C and then dried using the freeze dryer. Samples were ground and sieved as done for the extract above. Obtained powders were stored at 4°C until further analysis.

### 2.4 Determination of Total Phenolic Content and Encapsulation Efficiency

Encapsulation efficiency (EE) was determined as the ratio of the amount of phenolics in the core to the total amount of phenolics in encapsulates (Blagojević et al. 2022). The total phenolic content (TPC) in the samples was determined by the Folin–Ciocalteu method with gallic acid as a standard (Singleton et al. 1999). Encapsulated powder samples (0.3 g) were extracted with 3 mL of ethanol: methanol (50:50, v/v) for determination of total phenolic content (TPC) or ethanol:acetic acid: water (50:8:42, v/v/v) for total phenolic content on surface (SPC). An aliquot of 0.1 mL of each extract was mixed with 0.75 mL of Folin–Ciocalteu reagent (10%) and 0.6 mL of Na_2_CO_3_ solution (6% w/v). The mixtures were incubated at room temperature for 90 min. Absorbance of the mixtures was determined at 765 nm with a spectrophotometer (VWR, UV‐3100PC, Radnor, USA). A calibration curve with gallic acid was used and results were expressed as gallic acid equivalent (GAE). Equation (1) was used to determine the EE:

(1) $$\mathrm{EE}\;\left(\%\right)=\frac{\mathrm{TPC}-\mathrm{SPC}}{\mathrm{TPC}}x100$$

### 2.5 Determination of Antioxidant Activity

Antioxidant activity of the samples was analyzed by the cupric reducing antioxidant capacity (CUPRAC) (Apak et al. 2004). An aliquot of 100‐μL extract prepared for TPC analysis was mixed with 1 mL of 10 mM CuCl_2_.2H_2_O, 1 mL of 7.5 mM Neocuproine, and then 1 mL of 1 M NH_4_CH_3_CHOO (pH 7). The volume was completed to 4.1 mL with distilled water. The mixture was kept at room temperature for 30 min. The absorbance of the mixture was read at 450 nm with a spectrophotometer. Trolox was used as the standard for quantification.

### 2.6 Determination of Hesperidin Content

Hesperidin in the encapsulated samples was analyzed by RP‐HPLC (Waters 2695, W600 Waters, Milford, MA, USA) according to the method of Bino et al. (2005). Separation was carried out on a Supercosil LC‐18 25 cm x 4.60 mm, 5‐μm column (Sigma‐Aldrich, Steinheim, Germany). Two solvents, 0.1% (v/v) TFA with Milli‐Q water as A and 0.1% (v/v) TFA with acetonitrile as B, were used as mobile phases with a flow rate of 1 mL/min. A column temperature of 40°C and a sample volume of 10 μL were utilized. An external standard was used for the quantification of hesperidin.

### 2.7 Physicochemical Analyses of Encapsulates

The water activity (a_w_) was determined using a water activity meter (Protimeter, Meter House, England) at room temperature. The physical state of the encapsulated samples was determined by using an X‐ray diffractometer (Miniflex, Rigaku, Japan) according to the method of Papoutsis et al. (2018) with minor modifications. Radiation was generated at 40 kV. The scattering angle of 2θ was measured at a speed of 1°/min from 5° to 50°. The glass transition temperature of samples was determined by using a differential scanning calorimetry instrument (DSC Q10, TA Instruments, New Castle, USA) using 10 mg of sample. The sample was scanned from room temperature to −40°C at a rate of 10°C/min and then to 200°C at a rate of 10°C/min under nitrogen flow. The thermograms were analyzed by using Universal Analysis Software (TA Instruments, New Castle, USA).

The storage stability of encapsulated samples was determined by an accelerated storage test. One gram of each sample was spread in a Petri dish and then stored in a climatic chamber (HPP110, Memmert GmbH+Co, Schwabach, Germany) at a relative humidity of 75% and 40°C ± 1°C for 5 days.

### 2.8 Determination of ACE Inhibitory Activity

ACE inhibitory activity was determined by using an enzyme from rabbit lung (ACE, EC 3.4.15.1, Sigma Chemical Co., St. Louis, MO, USA) according to Martínez‐Alvarez et al. (2016) with minor modifications. A sample that contains 0.08 g encapsulated powder per mL was prepared in 100 mM borate buffer (pH 8.3) with 300 mM NaCl. An aliquot of 50 μL of the sample solution and 200 μL of 5 mM hippuryl‐L‐histidyl‐L‐leucine (HHL) were mixed and preincubated at 37°C for 15 min. The enzymatic reaction took place after the addition of 20 μL of ACE solution (100 mU in borate buffer) and incubation at 37°C for 60 min. The reaction was stopped by the addition of 250 μL of 1 N HCl. A control sample with 50 μL of borate buffer was prepared. The hippuric acid (HA) released was determined by RP‐HPLC using a C18 column (Tracer excel 120 ODSA 5 μm, Teknokroma, Barcelona, Spain). An injection volume of 10 μL and a flow rate of 0.8 mL/min were used. Water with 0.1% (v/v) TFA (Eluent A) and acetonitrile with 0.1% (v/v) TFA (Eluent B) were used as mobile phases. The column was eluted with 20% B for 5 min, followed by a linear gradient to 60% B for the next 15 min. Afterward, elution was maintained isocratically at 60% B for 4 min and then returned to the initial eluent composition of 20% B for 2 min. Peaks of HA and HHL were monitored at 228 nm. ACE inhibition was calculated by using Equation (2), where A_inhibitor_ and A_control_ express the area of the HA peak from the assays performed with and without ACE inhibitor, respectively.

(2) $$\text{Inhibition}\left(\%\right)=\left[1-\left(\;\frac{A_{\text{inhibitor}}}{A_{\text{control}}}\right)\right]x100$$

### 2.9 Determination of α‐Amylase Inhibitory Activity

α‐Amylase inhibition assay was carried out by using a sample of encapsulated powder at a concentration of 0.03 g/mL in 0.02 M sodium phosphate buffer (pH 6.9 with 6 mM sodium chloride) (Irondi et al. 2017). An aliquot of 500 μL of the sample solution and 500 μL 0.1 mg/mL α‐amylase solution (α‐amylase from porcine pancreas, Sigma Chemical Co., St. Louis, MO, USA) in the buffer were mixed and kept at 37°C for 10 min. A volume of 500 μL of 1% soluble starch solution in 0.02 M sodium phosphate buffer was added next. The reaction took place at 37°C for 15 min, and the reaction was stopped with 1.0 mL of DNS color reagent (1% 3,5‐dinitrosalicylic acid and 12% sodium potassium tartrate in 0.4 N NaOH). The mixture was held in a boiling water bath for 5 min and then cooled to room temperature. The absorbance of the mixture was measured at 540 nm. Percent inhibition was calculated by using Equation (2).

### 2.10 Statistical Analysis

Experimental samples were prepared in triplicate, and at least two samples from each replicate were analyzed. Effects of drying method, maltodextrin DE, and storage on the measured properties of the samples were evaluated by analysis of variance (ANOVA) and Tukey's multiple comparison test using the MINITAB software (MINITAB 18, Minitab Inc., Coventry, UK). A significance level of 0.05 was used in statistical analysis.

## 3 Results and Discussion

### 3.1 Encapsulation of Lemon Peel Extract

Phenolic compounds in lemon peel powder were extracted by enzyme‐assisted extraction and then freeze‐dried. The powder obtained in this way was sticky and difficult to handle. Encapsulation with maltodextrin and WPC and then drying rendered it nonsticky and free‐flowing. The final encapsulated powder contained 6.3% (w/w) lemon peel extract in dry matter.

Encapsulation efficiency was calculated based on the TPC of the samples before and after encapsulation. Drying method, maltodextrin DE, and their interaction significantly affected the encapsulation efficiency. FMD with maltodextrin 6DE resulted in the highest encapsulation efficiency. Greater drying temperatures in FMD may cause the carrier agent to form a wall structure faster, resulting in a greater encapsulation efficiency (Kanha et al. 2022). While maltodextrin DE affected efficiency in the case of FMD, no effect of DE was observed for FMFD. Maltodextrin 6DE contains less hydrolyzed starch with a higher average molecular weight compared to maltodextrin 19DE. Larger polymers in maltodextrin 6DE would entrap phenolic compounds more effectively by molecular entanglements. In addition, maltodextrin 6DE can protect phenolic compounds to a higher extent during drying by increasing the viscosity of the matrix and lowering molecular mobility, hence reducing the rate of degradation reactions (Roos 2020; Xiao et al. 2022).

**FIGURE 1:**
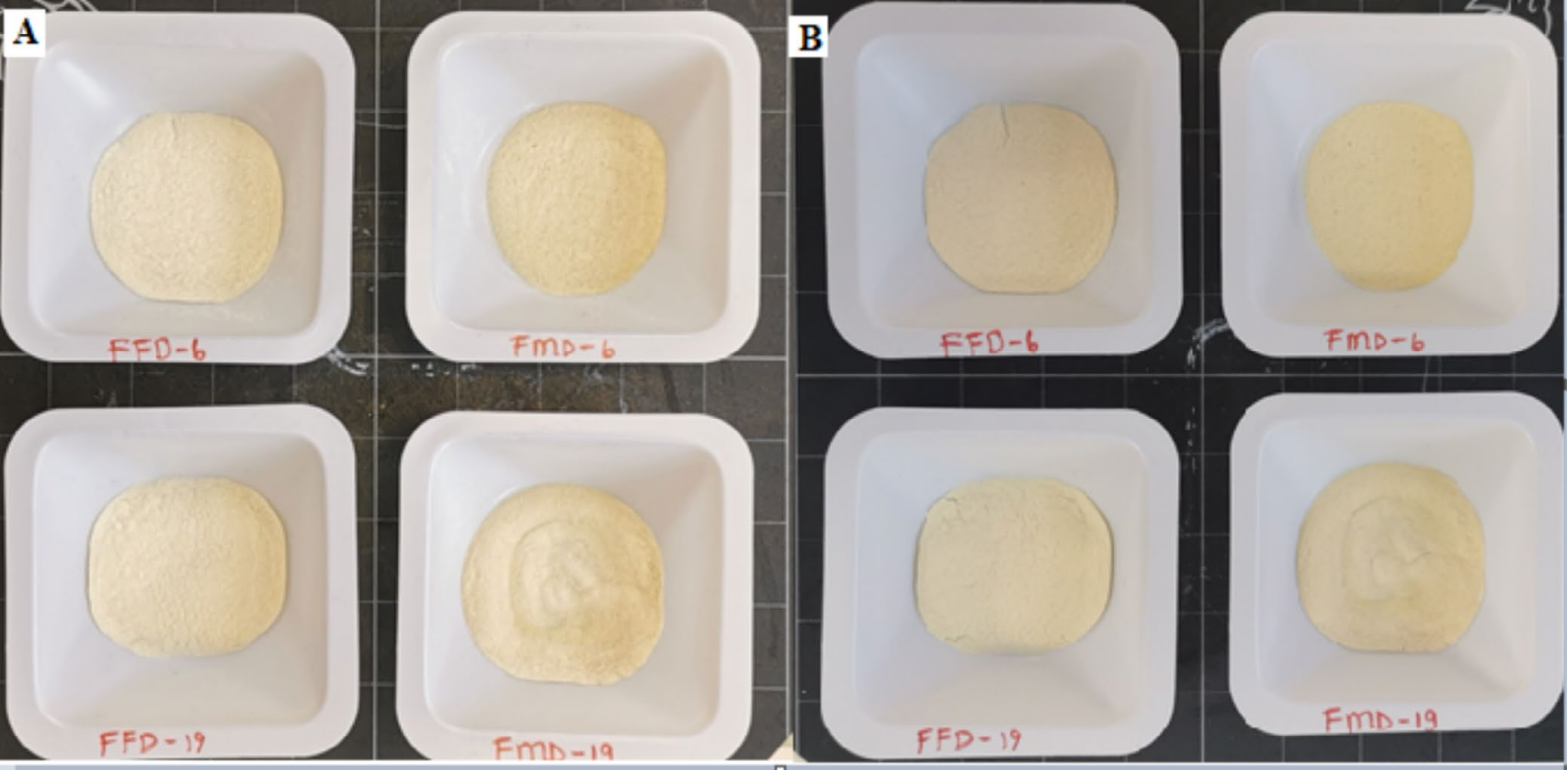
Images of the encapsulated lemon peel extracts with whey protein concentrate and different maltodextrins by different drying methods before (A) and after storage (B) at 40°C at 75% relative humidity for 5 days. FMFD‐6–FMFD‐19: Foam mat freeze dried with maltodextrin 6DE or 19DE, FMD‐6–FMD‐19: Foam mat dried with maltodextrin 6DE or 19DE.

When the color of the powders was examined before and after storage, it was clear that the FMFD preserved the color better while there was darkening in FMD treated samples possibly by Maillard reaction (Figure 1). It was also observed that the encapsulated samples with maltodextrin 19DE were slightly sticky and agglomerated after storage. The type of carrier agent used as well as the drying conditions have an impact on the hygroscopicity of the resulting powder. Maltodextrin with 19DE is more hygroscopic and reactive in the browning reaction due to the presence of more lower molecular weight and reducing starch fragments.

### 3.2 Physicochemical Properties of Encapsulated Lemon Peel Extracts

Water activity is a critical parameter influencing powder quality and stability. FMFD resulted in a lower water activity than FMD (Table 1). Sublimation under vacuum during freeze‐drying was more efficient in water removal than conventional drying. On the other hand, the formation of a crust and browning reaction might occur during FMD, resulting in higher water activity. In addition, maltodextrin with 19DE led to higher water activity by FMFD, but no effect of DE was observed for FMD. Maltodextrin with 19DE possibly would have more interactions with water molecules due to a higher number of lower molecular mass carbohydrates with more hydroxyl groups that can react with water (Zhu et al. 2022).

**TABLE 1:** Encapsulation efficiency and physicochemical properties of lemon peel extracts produced by using maltodextrin, whey protein concentrate, and different drying methods.

Drying method	Maltodextrin (DE)	Encapsulation efficiency (%)	Water activity (%)	Tg1 (°C)	Tg2 (°C)
Foam mat freeze‐drying	6	87.1 ± 1.5^b^	0.23 ± 2.80^c^	42.7 ± 6.3^a^	97.1 ± 0.2^a^
Foam mat drying	6	97.6 ± 0.7^a^	0.38 ± 0.00^a^	47.8 ± 6.5^a^	85.8 ± 2.1^b^
Foam mat freeze‐drying	19	88.7 ± 1.4^b^	0.28 ± 2.80^b^	47.5 ± 0.4^a^	86.5 ± 1.3^b^
Foam mat drying	19	90.1 ± 1.0^b^	0.34 ± 0.70^ab^	48.5 ± 1.0^a^	‐^nd^

*Note:* Mean ± SD (*n* = 3). Means marked with different letters (a, b, c) within a column are significantly different (*p* < 0.05).

Abbreviations: DE, Dextrose equivalent; nd, Not detected; Tg, Glass transition temperature.

**FIGURE 2:**
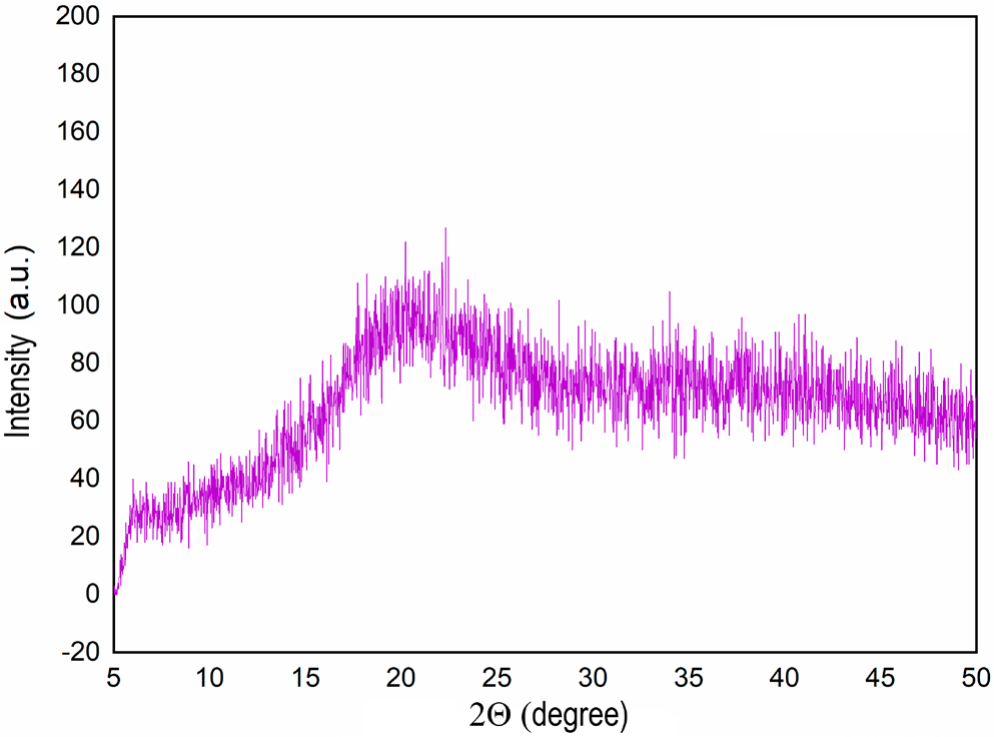
XRD pattern of the encapsulated foam mat freeze‐dried lemon peel extract with maltodextrin 6DE.

Stability of a powdered food material depends on the Tg of the matrix which determines its physical state during storage. If the Tg is higher than the storage temperature, the matrix remains in a glassy state in which molecular mobility is limited; hence rates of deterioration reactions such as oxidation are minimized. All the samples showed a similar XRD pattern that indicated they were in a glassy state (Figure 2). The glassy state matrix can preserve the bioactive compounds against heat and oxygen‐induced deterioration. There were two clear glass transition regions in the thermogram of encapsulated samples, except the sample treated with FMD and maltodextrin 19DE which showed only one Tg. The glass transition temperature around 43°C–48°C (Tg1) possibly belongs to WPC, and the glass transition temperature around 86°C–97°C (Tg2) was of maltodextrins (O'Donoghue et al. 2020; Siemons et al. 2020). Tg of maltodextrins is directly proportional to their molecular mass; hence, indirectly proportional to their DE value (Silalai and Roos 2011). The use of maltodextrin 6DE with FMFD resulted in the highest Tg2, which indicates that this sample would remain in the glassy state longer than the other samples. On the other hand, when FMD was applied with maltodextrin 6DE, a lower Tg2 was detected, possibly due to the higher water activity of this sample. Moisture content of a glassy matrix is inversely proportional to its Tg, and moisture uptake during storage can plasticize the matrix, reducing the Tg (Roos 2020). Change in the physical state of the matrix directly influences the stability of the core material at a rate proportional to the difference between Tg and storage temperature. When the storage temperature is equal to the Tg of the matrix, transition from glassy to rubbery state occurs, and increased molecular mobility accelerates the deterioration of the core material (Papoutsis et al. 2018). As a result, maltodextrin with a low DE value can more effectively protect the core material during storage.

### 3.3 Phenolic Content and Antioxidant Activity of Encapsulated Lemon Peel Extract

TPC and antioxidant activity of the lemon peel extract powder were 30.2 ± 0.46 mg GAE/g and 52.6 ± 7.0 mg TE/g, respectively. When TPC and antioxidant activity of the encapsulated samples were examined, they seemed to increase after encapsulation of the extract (Table 2). Unselective reaction of the Folin‐Ciocalteu phenol reagent used in the determination of TPC could enhance TPC values. Substances with a reducing power present in plant food extracts such as ascorbic acid, dehydroascorbic acid, and reducing sugars can react with the Folin–Ciocalteu reagent (Sánchez‐Rangel et al. 2013). Moreover, maltodextrins and WPC can contribute to TPC substantially as they include reducing sugars as well (Cantero et al. 2023). Although there is a strong association between phenolic content and antioxidant activity, ascorbic acid is a significant antioxidant in lemon and can add to the antioxidant capacity of the samples. Antioxidant activity of WPC and Maillard reaction products, especially in the samples dried with FMD, might also affect the antioxidant activity of the samples. Contrary to our results, Rashid et al. (2022) found nearly 70% and 67% decrease in TPC of pomegranate peel after encapsulation with maltodextrin, WPI alone, and maltodextrin‐WPI by freeze‐drying, respectively. Papoutsis et al. (2018) also found that TPC of a lemon by‐product decreased by 41% and 25% after encapsulation with maltodextrin‐carrageenan complex and maltodextrin‐soybean protein isolate complex by freeze‐drying, respectively. The use of WPC instead of WPI and the different methods used for antioxidant activity might be effective on the discrepancy between results from the studies. WPC contains more lactose, minerals, and other minor compounds besides whey proteins compared to WPI that might influence the TPC and antioxidant activity.

**TABLE 2:** Phenolic content and antioxidant activity of lemon peel extracts encapsulated with maltodextrin and WPC by different drying methods.

Drying method	Maltodextrin DE	Total TPC initial (mg GAE/g dry solid)	Total TPC after storage (mg GAE/g dry solid)	Surface TPC initial (mg GAE/g dry solid)	Surface TPC after storage (mg GAE/g dry solid)	CUPRAC initial (mg TE/g dry solid)	CUPRAC after storage (mg TE/g dry solid)
Foam mat freeze‐drying	6	6.63 ± 0.10^aA^	4.95 ± 0.03^aB^	0.85 ± 0.11^aA^	0.03 ± 0.01^aB^	17.6 ± 1.4^aA^	15.4 ± 0.9^aA^
Foam mat drying	6	4.59 ± 0.10^dA^	4.23 ± 0.2^bB^	0.11 ± 0.28^cA^	0.01 ± 0.10^bB^	11.6 ± 0.1^bA^	10.5 ± 0.2^bcB^
Foam mat freeze‐drying	19	5.38 ± 0.10^cA^	4.02 ± 0.1^bB^	0.61 ± 0.07^bA^	0.18 ± 0.03^bB^	13.4 ± 0.4^bA^	10.3 ± 0.3^cB^
Foam mat drying	19	5.87 ± 0.20^bA^	4.18 ± 0.1^bB^	0.61 ± 0.06^bA^	0.02 ± 0.02^bB^	13.3 ± 0.7^bA^	11.7 ± 0.4^bB^

*Note:* Mean ± SD (*n* = 3). Means with different superscript lowercase letters in columns and uppercase letters in rows are significantly different (*p* < 0.05).

Abbreviations: CUPRAC, Cupric reducing antioxidant capacity; GAE, gallic acid equivalents; TE, Trolox equivalent; TPC, Total phenolic content.

TPC and antioxidant activity of the samples were changed significantly by drying method, maltodextrin DE, storage, and the interactive effect of drying method and maltodextrin DE. The sample treated with FMFD and maltodextrin 6DE had the highest TPC among the encapsulated samples. Similar to our results, Gavahian et al. (2022) also found that when oven‐drying was replaced with freeze‐drying, the antioxidant capacity of *C. limon* (cv. Eureka) waste increased. Filiz (2023) showed that the drying of hawthorn by FMFD yielded greater TPC and antioxidant capacity values than those of the samples dried by FMD. A higher temperature of drying in FMD can degrade phenolic compounds in the samples. In addition, high heat treatment can cause the breakdown of cellular structures and the release of phenolic compounds such as phenolic acids bound to cell wall macromolecules. Thus, encapsulated phenolics bound to wall materials can be released and then degraded as a result of FMD carried out at a higher temperature compared to freeze‐drying (Kandasamy and Naveen 2022). Furthermore, the reduction in TPC can be due to the interactions between phenolic compounds and proteins. On the other hand, when maltodextrin with 19DE was used, FMD and FMFD yielded almost similar TPC and antioxidant activity. This might be related to more interactions of maltodextrin 19DE with phenolic compounds compared to maltodextrin 6DE.

The TPC and antioxidant activity of the samples were reduced after 5 days of storage at 75% relative humidity and 40°C. The sample treated with FMFD and maltodextrin 6DE had the highest TPC and antioxidant activity after storage. As a result of less damage during FMFD, this sample had more TPC and antioxidant activity at the beginning of storage. About 25% reduction in TPC and 12.5% reduction in antioxidant activity were observed after storage in this sample. A similar level of reduction took place in the samples treated with FMD or FMFD and maltodextrin 19DE, while the sample dried with FMD and maltodextrin 6DE had lesser reductions. The results imply that both wall materials and drying methods had an influence on the TPC and antioxidant activity. Water absorption by the matrix during storage causes the transition of the glassy matrix to a rubbery state; plasticization by water and microstructural collapse accelerate the degradation of the core material (Zhu et al. 2022).

### 3.4 Hesperidin Content of Encapsulated Lemon Peel Extracts

**FIGURE 3:**
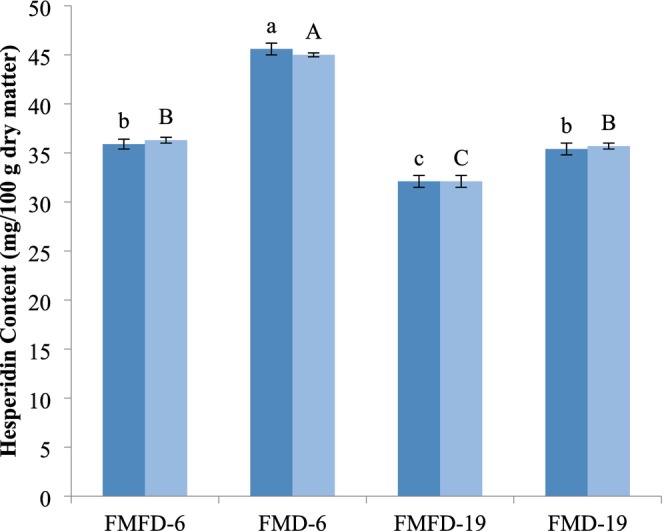
Hesperidin content of encapsulated lemon peel extracts with whey protein concentrate and different maltodextrins by different drying methods before (dark blue) and after (light blue) storage at 40°C at 75% relative humidity for 5 days. FMFD‐6–FMFD‐19: Foam mat freeze dried with maltodextrin 6DE or 19DE, FMD‐6–FMD‐19: Foam mat dried with maltodextrin 6DE or 19DE. Each value is expressed as mean ± SD (*n* = 3). Means marked with different letters are significantly different (*p* < 0.05).

Hesperidin is the major bioactive phenolic compound present in lemon peel (Alu'Datt et al. 2017). Therefore, the concentration of hesperidin was measured as a reference to determine the stability of phenolic compounds after encapsulation and storage (Figure 3). Hesperidin content of the samples was affected by drying method, maltodextrin DE, and their interactive effects. The highest hesperidin content was obtained in the sample treated with FMD and maltodextrin with 6DE. This sample also had the highest encapsulation efficiency that supported this finding. FMD treatment yielded higher hesperidin content regardless of maltodextrin DE.

There was no significant change in the concentration of hesperidin in the samples after storage, indicating that the encapsulation process was successful in protecting hesperidin. On the other hand, the trend in hesperidin was not in agreement with those observed in TPC and antioxidant activity. The sample treated with FMFD and maltodextrin 6E had the highest TPC and antioxidant activity, and there was a decline in TPC and antioxidant activity after storage. The foam structure, freezing, and long drying time might induce the degradation of hesperidin in the case of FMFD. The TPC and antioxidant activity of the samples depend on the phenolic profile, in addition to the matrix and process conditions (Gómez‐Mejía et al. 2019). Hesperidin, as a glycosylated flavonoid with a sugar moiety, is more stable than its aglycone hesperetin and the phenolic acids present in the lemon peel extract. Therefore, there could be degradations in the other phenolic compounds during the storage time that cause reductions in TPC and antioxidant activity.

### 3.5 ACE Inhibitory Activity of Encapsulated Lemon Peel Extracts

**FIGURE 4:**
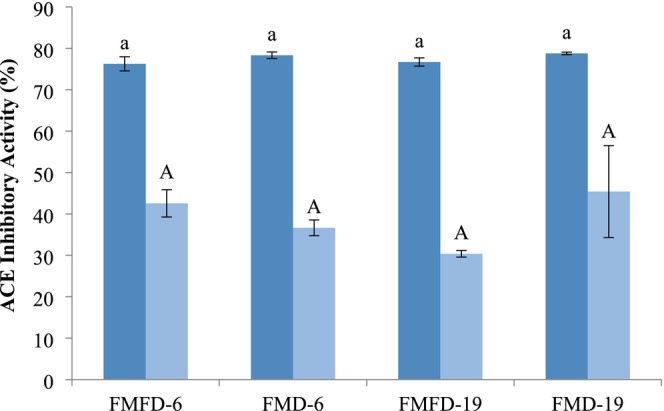
ACE inhibitory activity of lemon peel extracts encapsulated with whey protein concentrate and maltodextrin by different drying methods before (dark blue) and after (light blue) storage at 40°C at 75% relative humidity for 5 days. FMFD‐6–FMFD‐19: Foam mat freeze dried with maltodextrin 6DE or 19DE, FMD‐6–FMD‐19: Foam mat dried with maltodextrin 6DE or 19DE. Each value is expressed as mean ± SD (*n* = 3). Means marked with different letters are significantly different (*p* < 0.05).

ACE inhibitory activity of the lemon peel extract was 53.2% ± 1.2% at the concentration used for the encapsulation. After encapsulation with the wall materials, ACE inhibitory activity ranged from 76.7% to 78.3% which showed that wall materials also contributed to this activity (Figure 4). ACE inhibition by the samples was not influenced by the drying method and maltodextrin DE. ACE inhibitory activity of the lemon peel extract has been found to be mostly associated with polyphenols, hesperidin and its aglycone hesperetin being the major ones (Durmus and Kilic‐Akyilmaz 2023; Ruviaro et al. 2019). In addition, ascorbic acid present in the samples can contribute to the ACE inhibitory activity of the samples as well (Ivanov et al. 2021; Ruviaro, Durmus and Kilic‐Akyilmaz 2023). Moreover, Khajuria et al. (2020) reported a synergism between phenols, flavonoids, and ascorbic acid in vitro ACE inhibitory activity of pineapple extract. Whey proteins also possess ACE inhibitory activity, which would contribute to the level of inhibition observed in the samples (Olvera‐Rosales et al. 2023). In addition, products of the Maillard reaction, such as melanoidins, that can occur during drying, were shown to have ACE inhibitory activity in the range of 28.7%–64.3% depending on the dosage (Rufián‐Henares and Morales 2007). Bioactivity of melanoidins was attributed to their metal‐chelating or reducing activities since ACE is a Zn‐dependent enzyme.

ACE inhibitory activity of the encapsulated samples declined to less than 45% under adverse storage conditions with no significant difference between them. This trend was correlated with the reductions in TPC and antioxidant activity, although the level of reductions is not of the same magnitude. Polyphenols are unstable and extremely susceptible to degradation by oxygen and metal ions during storage, resulting in changes in their structures and a decrease in their biological activities. In addition, possible interactions between whey proteins and phenolics during storage may have caused a decrease in the ACE inhibitory activity of both components (Alu'Datt et al. 2017). Moreover, bound Maillard reaction products and melanoidins were reported to have a lower activity compared to free ones (Rufián‐Henares and Morales 2007). As a result of the complex composition of the encapsulated samples, the effects of the drying method and the physicochemical properties of the samples on ACE inhibitory activity were not clear.

### 3.6 α‐Amylase Inhibitory Activity of Encapsulated Lemon Peel Extracts

**FIGURE 5:**
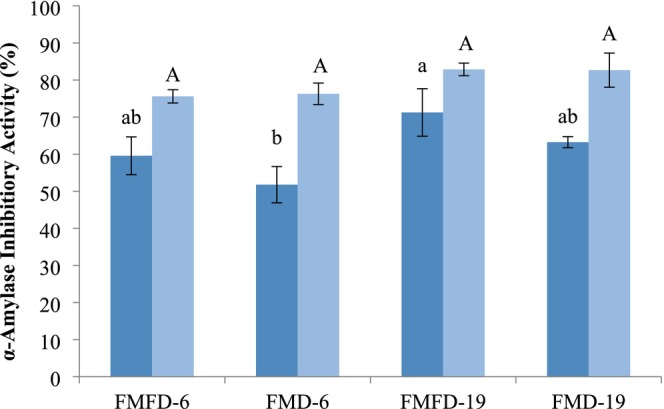
α‐Amylase inhibitory activity of encapsulated lemon peel extracts with whey protein concentrate and different maltodextrins by different drying methods before (dark blue) and after (light blue) storage at 40°C at 75% relative humidity for 5 days. FMFD‐6–FMFD‐19: Foam mat freeze dried with maltodextrin 6DE or 19DE, FMD‐6–FMD‐19: Foam mat dried with maltodextrin 6DE or 19DE. Each value is expressed as mean ± SD (*n* = 3). Means marked with different letters are significantly different (*p* < 0.05).

α‐Amylase inhibitory activity of the lemon peel extract at the concentration present in the encapsulated samples was measured as 9.3% ± 0.9%. Encapsulated samples exhibited inhibition in the range of 51.79%–71.26%, which proves that the wall materials added to the samples were also inhibitory to this enzyme (Figure 5). Drying method, DE of maltodextrin, and storage significantly affected α‐amylase inhibitory activity. Using FMFD instead of FMD resulted in a higher α‐amylase inhibitory activity, most likely due to the low temperature applied during drying, preserving the bioactive components better. The sample containing maltodextrin with 19DE showed higher α‐amylase inhibitory activity than the one with 6DE when the same method of drying was applied. The activity of α‐amylase is determined based on the hydrolysis of starch into lower molecular weight fragments with reducing ability. Maltodextrins are also starch hydrolysates, and maltodextrin with 19 DE has a higher concentration of reducing sugars and oligosaccharides. Hence, these inherent components of maltodextrins can inhibit α‐amylase by product inhibition, the extent of which will be enhanced in the case of maltodextrin with 19 DE. Besides maltodextrin, whey proteins have been shown to exert an inhibitory effect on α‐amylase (Baba et al. 2021). Moreover, interactions between whey proteins and phenolic compounds can modify the bioactivity of phenolic compounds. Li et al. (2023) reported that the binding of whey proteins and phenolic compounds caused a reduction in the competitive inhibition of phenolic acids on α‐amylase. Therefore, the bioactivity determined for an encapsulated phenolic extract will be affected by the action of phenolic and other components in the extract as well as the wall materials used and their interactions.

α‐Amylase inhibitory activity increased after storage under adverse conditions. This might be related to the degradation of the phenolic–protein complexes that can promote the inhibitory activity of the phenolic compounds. In addition, the Maillard reaction that can occur between the components of the encapsulated powder during storage can influence the activity. End products of the Maillard reaction have been shown to exert antioxidant and α‐glucosidase inhibitory activities (Hwang et al. 2011).

## 4 Conclusions

Lemon peel phenolic extract obtained by enzyme‐assisted extraction was encapsulated by using maltodextrin and WPC for enhancing storage stability. Maltodextrin with 6DE was a better wall material than maltodextrin with 19DE when used along with WPC. The sample treated with FMFD and maltodextrin 6DE had the highest Tg, TPC, and antioxidant activity before and after storage. FMD‐treated sample with maltodextrin 6DE yielded the highest encapsulation efficiency and hesperidin content. Both FMD and FMFD may be used as drying methods in encapsulation. However, since FMD is more practical with lower equipment and energy costs, and a shorter duration, it could be a good option for encapsulation processes.

Encapsulated lemon peel extracts exerted significant levels of ACE and α‐amylase inhibitory activities in in vitro tests. The trends observed in the bioactivities indicated that the wall materials, in addition to the lemon peel extract, had an influence on the inhibition of the enzyme activities. Therefore, it is recommended that the impact of the wall materials on a particular bioactivity is also considered in encapsulation applications.

This study showed a potential for upcycling lemon peel as an antioxidant, antihypertensive, and antidiabetic functional food ingredient, nutraceutical, or pharmaceutical toward the improvement of public health. However, long‐term storage studies under normal storage conditions should be conducted to determine the actual shelf life of the encapsulated extract. In addition, the application and stability of this extract as an ingredient in different food products can be evaluated.

## 5 Practical Applications

Lemon peel waste is a rich source of bioactive phenolic compounds that can be upcycled to a functional food ingredient, nutraceutical, or pharmaceutical. However, phenolic compounds should be protected from environmental conditions to maintain their bioactivity during storage. This study showed that encapsulation of lemon peel phenolic extract by using maltodextrin 6DE and whey protein concentrate and foam mat drying can result in a bioactive product with antihypertensive and antidiabetic activity. Both foam mat drying and foam mat freeze drying are applicable for the process, while foam mat drying can be preferred for reducing equipment cost, energy, and process time.

## Author Contributions

**Nihal Durmus:** conceptualization (equal), data curation (equal), formal analysis (lead), investigation (lead), methodology (equal), writing – original draft (equal), writing – review and editing (equal). **Meral Kilic‐Akyilmaz:** conceptualization (equal), data curation (equal), funding acquisition (lead), methodology (equal), project administration (lead), resources (lead), supervision (lead), writing – review and editing (equal).

## Ethics Statement

The authors have nothing to report.

## Conflicts of Interest

The authors declare no conflicts of interest.

## Data Availability

The data will be made available upon reasonable request.
